# H19 and IGF2 imprinting from embryogenesis to oncogenesis

**DOI:** 10.3389/fcell.2026.1698015

**Published:** 2026-03-11

**Authors:** Bella Ortega, Rida Saeed, Sloan White, Patrick Tajanlangit, G. Ian Gallicano

**Affiliations:** Georgetown University Medical Center, Washington, DC, United States

**Keywords:** cancer epigenetics, DNA methylation, embryonic development, epigenetic regulation, genomic imprinting, H19/IGF2 locus, loss of imprinting (LOI), noncoding RNA

## Abstract

The imprinted *H19*/*IGF2* locus is critical for fetal development and, when dysregulated, contributes to tumorigenesis. This review examines the mechanisms regulating imprinting through DNA methyltransferases, alongside shared signaling pathways, such as PI3K/AKT, that operate across both embryonic development and tumorigenesis. Parent-of-origin methylation at this locus coordinates differential gene activity: *IGF2* promotes organogenesis and placental angiogenesis through mitogenic signaling, while *H19*, a long non-coding RNA that serves as a precursor for miR-675-5p, prevents fetal overgrowth, regulates trophoblast invasion, and modulates epithelial-to-mesenchymal transitions (EMT). Loss of imprinting (LOI) at this locus disrupts normal gene expression, contributing to the development of cancer and imprinting disorders. Overexpression of *H19* activates the PI3K/AKT pathway and silences tumor suppressor genes such as *let-7b* through sponging target genes and subsequently inducing EMT. While LOI of *IGF2* leads to cancer through upregulation of the *Wnt3* pathway. ​​*H19* and *IGF2* play opposing yet coordinated roles in embryogenesis, with *IGF2* promoting proliferation and *H19* restricting overgrowth, a balance essential for proper tissue development. When epigenetically disrupted, it enables tumors to reactivate developmental pathways that drive unchecked growth, EMT, metastasis, and resistance to apoptosis. Controlling the balance of imprinting of the *H19*/*IGF2* locus presents a promising mechanism for future therapies.

## Introduction

The epigenome is the pattern of genetic modifications, such as DNA and histone modifications, that determine the expression of genes. Genomic imprinting occurs when there is a difference in the epigenetic modifications between parental alleles, leading to differing levels of expression across maternal and paternal copies of the same gene. Some of the earliest identified imprinted genes, *H19* and *IGF2*, are located on chromosome 11q15.5 and share a common imprinting control region (ICR), called H19DMR (Chang et al., 2022; Soejima and Higashimoto, 2013). Together, these genes play significant roles in fetal development, including regulating epithelial-to-mesenchymal transition (EMT), the process by which epithelial cells transform into mesenchymal stem cells.

Loss of imprinting (LOI) occurs when there is a lack of normal epigenetic modifications in imprinted genes. During fetal development, LOI of *H19* and *IGF2* can result in serious developmental disorders. For example, a subgroup of Beckwith-Wiedemann syndrome (BWS) is caused by hypermethylation of the ICR for *H19* and *IGF2* on the maternal allele, leading to biallelic *IGF2* expression and silencing of both *H19* alleles, ultimately resulting in fetal overgrowth (Chang et al., 2022). In addition to presenting with fetal overgrowth, lateralized overgrowth, and abdominal wall defects, BWS is associated with embryonic tumors, such as Wilm’s tumor, suggesting a connection between *H19/IGF2* LOI and tumorogenesis (Mussa and Kalish, 2023; Soejima and Higashimoto, 2013). In contrast, Silver-Russell Syndrome (SRS) occurs due to hypomethylation of ICR on the paternal allele, resulting in the biallelic expression of *H19* and significant reduction or silencing of *IGF2* expression, ultimately leading to fetal growth restriction (Chang et al., 2022).

Conversely, neoplastic growth can occur when there is LOI of *H19* and *IGF2* during adulthood. H19 and *IGF2* are believed to be heavily involved in EMT, a cellular mechanism hijacked during tumor growth and metastasis (Xu et al., 2021). By understanding their mechanistic roles in fetal development and cellular proliferation, researchers can develop targeted therapies to address the loss of *H19* and *IGF2* imprinting seen in many cancers.

## Mechanisms of imprinting at the *H19*/*IGF2* locus

As a highly regulated and reciprocally expressed locus, the *H19*/*IGF2* locus is integral to mammalian development. *IGF2*, which encodes for insulin-like growth factor 2, is paternally expressed and is demonstrated to possess strong mitogenic effects (Liao J et al., 2023). Conversely, *H19* is maternally expressed, and it encodes for a long non-coding RNA (lncRNA) with both growth-suppressive and regulatory properties (Liao W et al., 2023).

The reciprocal expression of this locus is highly regulated by the methylation state of the Imprinting Control Region 1 (ICR1), which controls the accessibility of the shared downstream enhancers for the *H19*/*IGF2* locus (Xie et al., 2024). The ICR1 is located upstream of *H19* and functions in a parent-of-origin manner (Liao J et al., 2023). On the maternal allele, ICR1 is hypomethylated, causing the CCCTC-binding factor (CTCF) to bind and block the downstream enhancer from accessing the *IGF2* promoter, resulting in the silencing of *IGF2* while maintaining *H19* expression (Liao W et al., 2023). On the paternal allele, ICR1’s hypermethylated state inhibits CTCF binding, allowing for *IGF2* activation and *H19* silencing (Liao J et al., 2023; Xie et al., 2024).

### Role of DNA methyltransferases (DNMTs) in maintaining imprinting

The maintenance and establishment of genomic imprinting methylation patterns at the *H19*/*IGF2* locus are regulated by DNA Methyltransferases (DNMTs) (Han et al., 2023). A study comparing *H19* promoter methylation levels and DNMT expression between patients who experienced spontaneous abortion (SA) following *in vitro* fertilization embryo transfer and patients with healthy early pregnancies found significantly reduced DNMT3A and DNMT3B mRNA levels in the chorionic villus of SA patients, while DNMT1 expression remained unchanged. (Han et al., 2023). These findings are critical to understanding imprinting at this locus, as DNMT1 primarily functions during DNA replication to copy existing methylation patterns onto newly synthesized DNA strands (Han et al., 2023). Meanwhile, DNMT3A and DNMT3B establish *de novo* methylation patterns during early embryonic development and gametogenesis, providing a foundational epigenetic blueprint (Han et al., 2023). Correspondingly, *H19* in SA patients was hypomethylated, suggesting a correlation between reduced DNMT3A and DNMT3B activity, altered *H19* methylation, and increased risk of pregnancy loss (Han et al., 2023). Therefore, these findings reveal that precise temporal coordination between *de novo* and maintenance of methylation is essential for maintaining imprinting stability from early embryogenesis through somatic differentiation (Li et al., 1993; Okano et al., 1999; Bourc’his and Bestor, 2004; Han et al., 2023).

Building on this, early knockout studies in mice provided the first mechanistic insight into how DNMTs preserve genomic imprinting. Li et al. (1993) demonstrated that complete loss of DNMT1 results in global demethylation, loss of imprinting, and embryonic lethality, underscoring its essential role in maintaining methylation inheritance during DNA replication. Subsequently, Okano et al. (1999) then revealed that DNMT3A and DNMT3B establish *de novo* methylation marks during gametogenesis and early embryogenesis, creating the parental methylation asymmetry at the H19/IGF2 ICR. These findings collectively defined the distinct but complementary roles of DNMT1 and DNMT3 enzymes in establishing and perpetuating parent-of-origin methylation.

In parallel with DNMT activity, a network of transcriptional, chromatin, and RNA-based regulators ensures proper targeting and stability of the H19/IGF2 ICR1. The insulator protein CTCF, in complex with the cohesin complex subunits RAD21 and structural maintenance of chromosomes 3 (SMC3), binds the unmethylated maternal ICR1 to form long-range chromatin loops that block enhancer access to IGF2. Methylation of the paternal allele prevents CTCF–cohesin binding, thereby permitting IGF2 activation (Bell and Felsenfeld, 2000; Kurukuti et al., 2006). Germline-specific transcription factors also guide the establishment of methylation asymmetry. The CCCTC-binding factor–like (CTCFL) protein, also known as BORIS (Brother of the Regulator of Imprinted Sites), is a testis-specific paralog of CTCF that functions during spermatogenesis to recruit the *de novo* methyltransferases DNMT3A and DNMT3B to the paternal ICR1, initiating the hypermethylated paternal state (Nishana et al., 2020).

Additional transcriptional regulators reinforce locus-specific chromatin architecture. The Kruppel-like factor 4 (KLF4) transcription factor promotes chromatin accessibility during early embryogenesis, facilitating proper imprint establishment (Ye et al., 2018). In contrast, Yin Yang 1 (YY1) functions as a repressor by recruiting the Polycomb Repressive Complex 2 (PRC2), whose catalytic subunit enhancer of zeste homolog 2 (EZH2) deposits trimethylation on histone H3 at lysine 27 (H3K27me3), a repressive histone modification that compacts chromatin and stabilizes long-term silencing of H19 on the paternal allele (Kim et al., 2003).

Non-coding transcripts provide an additional layer of regulation: IGF2-AS stabilizes IGF2 mRNA (Jin, 2008), intragenic miR-483 modulates IGF1 signaling by repressing IGF1 (Sandovici et al., 2024) and H19-derived miR-675 restricts trophoblast invasion by targeting IGF1R (Keniry et al., 2012). Collectively, these non-coding RNAs modulate IGF2/H19 pathway activity and act parallel to DNA methylation to fine-tune gene expression at the H19/IGF2 locus. When disrupted, this cooperative network contributes to pathological ICR1 hypermethylation and loss of imprinting, phenomena characteristic of overgrowth syndromes and cancers such as Wilms tumor and hepatoblastoma.

In addition to these methyltransferases, UHRF1 is a ubiquitin-like protein that binds hemi-methylated DNA and recruits DNMT1 to replication forks, ensuring faithful propagation of methylation patterns across cell divisions (Bostick et al., 2007). Studies further revealed that UHRF1 functions as a molecular bridge by recognizing hemi-methylated CpG dinucleotides and histone H3K9me2, thereby linking DNA and histone methylation during replication to maintain local chromatin states (Sharif et al., 2007).

Together, UHRF1 and DNMT1 ensure that DNA methylation patterns are faithfully copied during every cell division, maintaining stable imprinting across generations of cells. At the same time, histone-modifying enzymes such as G9a (also known as EHMT2) and the zinc-finger protein ZFP57 work alongside DNMTs in a reinforcing feedback loop (Quenneville et al., 2011). This coordination preserves the repressive histone mark H3K9me2 at imprinted regions, which keeps chromatin compact and prevents enhancers from inappropriately activating normally silent genes.

Aberrant DNMT3A/3B activity or depletion of UHRF1 disrupts the maternal H19/IGF2 boundary, resulting in LOI and biallelic IGF2 expression, as seen in cancers such as Wilms tumor, hepatoblastoma, and colorectal carcinoma (Ren, 2022). These cancers display focal hypomethylation within ICR1, accompanied by chromatin relaxation and reactivation of fetal growth programs. The DNMT-mediated epigenetic machinery illustrates the evolutionary continuity between developmental imprint maintenance and tumor suppression, highlighting that pathways safeguarding embryonic epigenetic stability also constrain malignant reprogramming in somatic cells.

### Timing and regulation of *H19*/*IGF2* expression during development

Not only do the *Igf2* and *H19* genes possess reciprocal expression, but they also differ in their postnatal activity. *Igf2* is highly expressed during embryonic and fetal development and largely decreases after birth, with some continued expression in stem cells. Igf2 was found to be expressed in mouse neural stem cells to regulate neurogenesis in the subventricular and subgranular zone of the hippocampus (Ferrón et al., 2015). Intestinal stem cells of adult mice also show continued Igf2 expression with Igf2 deletion resulting in stem cell loss in the crypts of Lieberkühn (Ziegler et al., 2019). Contrastingly *H19* is also predominantly active embryologically, with postnatal expression largely restricted tissues such as skeletal and cardiac muscle in mice (Park et al., 2021). Interestingly, in blastocysts, *IGF2* expression is biallelic and shifts to monoallelic paternal expression after implantation (Lerchner and Barlow, 1997). While the *H19*’s differentially methylated region (DMR) is methylated in sperm but not in oocytes. This differential methylation is maintained during all stages of development, excluding germ cells, as this methylation process must be recreated *de novo* (Lerchner and Barlow, 1997).

### Developmental functions of *IGF2*

*IGF2* functions as a potent and critical mitogen during embryonic development and contributes to the cellular proliferation and differentiation of the fetus and placenta. It binds to its receptors—insulin-like growth factor 1 receptor (IGF1R), insulin receptor (INSR), and IGF1R/INSR hybrid receptors—and subsequently activates intracellular signaling cascades via the PI3K/AKT and MAPK/ERK pathways (Wang et al., 2018). These pathways promote cellular survival, proliferation, protein synthesis, and growth, and are therefore critical for somatic and placental maturation.

Beyond its role in fetal growth, IGF2 is pivotal to organogenesis, coordinating cell proliferation, differentiation, and metabolic maturation across organs such as the liver, adipose tissue, pancreas, skeletal muscle, and central nervous system, as seen in Table 1 (Sélénou et al., 2022; Wang et al., 2018).

**TABLE 1:** Summary of IGF2’s roles in embryonic organ development.

Organ/Tissue	Role of IGF2	Mechanism/Pathway involved
Liver	Promotes glycogen synthesis and hepatocyte proliferation	PI3K/AKT signaling; acts via autocrine/paracrine loops
Adipose (subcutaneous)	Stimulates preadipocyte differentiation	Enhances insulin signaling; increases GLUT4 expression
Adipose (visceral)	Inhibits preadipocyte differentiation	Downregulates INSR-A and GLUT4
Pancreas (mesenchyme)	Promotes β-cell proliferation and mesenchymal expansion	Paracrine signaling during fetal development
Skeletal muscle	Drives myoblast differentiation from mesodermal precursors	Upregulates MyoD via PI3K/AKT
Central nervous system	Supports neurogenesis, hippocampal plasticity, memory consolidation, and learning	IGF2R and AMPA receptor signaling

This table outlines the organ-specific developmental functions of IGF2 and the distinct molecular pathways involved. IGF2 contributes to diverse developmental processes, including metabolic programming (liver, adipose), endocrine differentiation (pancreas), structural maturation (skeletal muscle), and neurodevelopment (central nervous system), primarily through signaling cascades like PI3K/AKT, and IGF2R-mediated pathways. The tissue-specific effects of IGF2 reinforce IGF2’s role as a multifaceted growth factor essential for fetal organogenesis and maturation. Abbreviations: INSR-A, Insulin Receptor Isoform A; MyoD, myogenic differentiation factor; IGF2R, Insulin-like Growth Factor 2 Receptor (Sélénou et al., 2022; Wang et al., 2018).

Of particular note is *IGF2*’s function in placental development. Within the labyrinthine zone, the site of maternal-fetal nutrient exchange, conditional *IGF2* knockout models show reduced fetal capillary density, increased endothelial apoptosis, and decreased proliferation, ultimately resulting in compromised maternal–fetal nutrient and oxygen exchange, leading to fetal growth restriction (Sandovici et al., 2022). Moreover, *IGF2* signaling promotes the expression of key trophoblast differentiation markers, including Gcm1 and Synb (Sandovici et al., 2022). These findings reveal the *IGF2*’s vital role in activating its receptor pathway to drive angiogenesis and promote trophoblast differentiation, which are essential for syncytiotrophoblast formation and efficient placental function.

### Developmental functions of *H19*

To balance out *IGF2* mitogenic properties, the *H19* gene possesses growth suppressive properties during embryonic and placental development and is downregulated postnatally (Liao W et al., 2023; Zhang et al., 2018). Although *H19* is a long non-coding RNA*, it contains an* embedded microRNA, miR-675-5p, within its first exon (Ogoyama et al., 2021). Post-transcriptionally, miR-675-5p is excised from the H19 transcript via a canonical microRNA biogenesis pathway (Ogoyama et al., 2021). After initial processing in the nucleus, the miR-675-5p precursor is exported to the cytoplasm, where it is further cleaved into its mature form. The mature miRNA is then incorporated into the RNA-induced silencing complex (RISC), enabling it to bind complementary target mRNAs and suppress their expression (Suzuki, 2023).

Once active, the mature miR-675-5p regulates the balance between trophoblast proliferation and invasion (Ogoyama et al., 2021). RNA sequencing of first-trimester extravillous trophoblast (EVT) cells reveals that both *H19* and miR-675-5p are highly expressed in invasive cells compared to non-invasive trophoblast cells (Ogoyama et al., 2021). Also, overexpression of miR-675-5p in EVT cells significantly increases EVT invasiveness, which is critical for maternal spiral artery remodeling. This effect is driven by suppression of the transcription factor GATA2, as reduced expression of GATA2 subsequently leads to an upregulation of Metalloproteinase-13 (MMP13) and Matrix Metalloproteinase-14 (MMP14) (Ogoyama et al., 2021). MMP13 and MMP14 are critical enzymes in spiral artery remodeling, as they degrade the extracellular matrix, thereby promoting further trophoblast invasion into the uterus. The knockdown of either MMP13 or MMP14 significantly reduces trophoblast invasiveness, confirming their essential roles downstream of the *H19/*miR-675-5p pathway (Ogoyama et al., 2021). Furthermore, GATA2 suppression also resulted in upregulation of H19, enhancing miR-675-5p production (Ogoyama et al., 2021). This suggests a positive feedback loop in which miR-675-5p reinforces its own expression via *GATA2* downregulation, allowing for the persistence of invasive properties necessary for proper spiral artery remodeling and early placental development (Table 2).

**TABLE 2:** H19 and miR-675 in embryonic and placental development.

Organ/Tissue	Role of H19/miR-675	Mechanism/Pathway
Placenta (EVT/spiral artery remodeling)	Tunes balance of invasion versus restraint during placentation	H19 → miR-675-5p suppresses GATA2, upregulates MMP13/MMP14, promotes EVT invasion; intersects PI3K/AKT/ERK signaling
Fetal growth regulation	Restricts overgrowth; counters IGF2 mitogenic drive	Maternal H19 dosage and imprinting at ICR1; growth-suppressive lncRNA functions
Heart and skeletal muscle (postnatal persistence)	Tissue-restricted maintenance of H19 expression	H19 mis-expression in LOI models is associated with cardiac pathology; dosage sensitivity highlighted in loss-of-imprinting mouse models

This table summarizes developmental functions discussed in the H19 sections, emphasizing miR-675-5p as an effector of trophoblast invasion and the dosage-sensitive growth restraint of H19 (Ogoyama et al., 2021; Park et al., 2021).

### Epithelial-to-mesenchymal transition in embryonic development

Interestingly, the MMP-mediated ECM degradation seen in EVT is also observed during EMT. Since EVTs originate from cytotrophoblasts, which are epithelial in nature, and become invasive, motile trophoblasts, this process can arguably be analogous to cellular morphogenesis seen in EMT. While literature on the direct influence of the *H19*/*IGF2* locus on embryological EMT is limited, certain signaling pathways between EMT and EVT do converge.

Both *H19* and *IGF2* independently regulate PI3K/AKT signaling, which plays an important role in the cellular transitions of both EVT and EMT. *H19* represses EMT-restrictive GATA2, and *IGF2* signaling directly activates these pathways via binding to transmembrane tyrosine kinase receptors (Ogoyama et al., 2021).

Moreover, in EVT, trophoblast migration is driven by granulocyte colony-stimulating factor (G-CSF) secreted by alternatively activated M2 macrophages, which activates PI3K/AKT signaling; inhibition of this pathway significantly impairs trophoblast invasiveness, proving its functional necessity (Ding et al., 2021). Similarly, in EMT, PI3K/AKT signaling proves critical for driving the transition from an adhesive, polarized epithelial state to a more motile, invasive mesenchymal phenotype, as seen in early embryonic processes such as gastrulation. Inhibition of this signaling pathway has been shown to impair inner cell mass (ICM) cells from developing into epiblasts, which give rise to all three germ layers that are essential for the formation of all tissues and organs (Geiselmann et al., 2025). Beyond this canonical PI3K/AKT axis, IGF2 signaling also activates the MAPK/ERK cascade, which exerts complementary but distinct control over EMT transcriptional programs.

While the PI3K/AKT pathway is well established as a driver of IGF2-mediated EMT, the parallel MAPK/ERK cascade also plays a crucial and complementary role in orchestrating transcriptional reprogramming during both embryogenesis and tumorigenesis. Upon IGF2 binding to IGF1R, INSR-A, or hybrid IGF1R/IR-A receptors, Ras activation triggers a sequential phosphorylation cascade through Raf-1, MEK1/2, and ERK1/2. Activated ERK1/2 translocates to the nucleus, where it induces transcription of canonical EMT regulators including SNAIL (SNAI1), SLUG (SNAI2), TWIST1/2, and ZEB1/2 (Lamouille et al., 2014). These transcription factors repress epithelial markers such as E-cadherin (CDH1) and activate mesenchymal genes (N-cadherin, Vimentin, Fibronectin), thereby promoting loss of adhesion and acquisition of motility.

Mechanistically, IGF2 signaling enhances ERK activation through autocrine and paracrine stimulation of IGF1R/INSR-A dimers, amplifying downstream phosphorylation of ELK1 and c-FOS that cooperate with AP-1 to promote SNAIL and TWIST transcription (Zhao et al., 2016). This provides a direct molecular route from IGF2 engagement to the induction of EMT master regulators. Furthermore, H19, through its encoded miR-675, modulates this axis by targeting IGF1R, creating a feedback circuit that fine-tunes ERK signaling intensity (Keniry et al., 2012). During times in which H19 expression is elevated, such as in trophoblast differentiation, this feedback attenuates and stabilizes ERK-driven motility to prevent excessive invasion; conversely, in cancers where H19 is epigenetically repressed, loss of this regulatory restraint leads to sustained ERK signaling and pathological EMT.

In developmental contexts, temporally regulated ERK activation downstream of IGF2 supports coordinated cell migration and tissue morphogenesis, such as during gastrulation and neural crest delamination. In oncogenesis, chronic and dysregulated ERK activity reinstates this embryonic migratory program, promoting basement membrane degradation, matrix remodeling, and metastatic dissemination. Together, the PI3K/AKT and MAPK/ERK pathways represent dual downstream arms of IGF2/H19 signaling that converge on EMT effectors, providing a mechanistic bridge between developmental plasticity and malignant progression. This expanded integration highlights the shared molecular logic that underlies morphogenesis and metastasis, illustrating how developmental signaling hierarchies are repurposed during oncogenic transformation.

Although the *H19*/*IGF2* locus may not directly initiate EMT, its shared molecular machinery with EVT’s PI3K/AKT signaling pathway illustrates a convergent role in cellular migration, plasticity, and lineage specification during development. The *H19*/*IGF2* locus plays a pivotal role in regulating embryonic growth, placental development, and tissue-specific differentiation. Through highly regulated imprinting mechanisms, *IGF2* promotes cellular proliferation and organogenesis, while *H19* functions to restrict overgrowth and modulate trophoblast invasion.

### *H19* misregulation in cancer

*H19* was originally identified as an imprinted gene expressed maternally during embryogenesis that is typically silenced after birth. However, abnormal re-expression of *H19* due to LOI has been observed in various types of cancer where it contributes to tumor growth, metastasis, and chemoresistance. While *H19* is typically an oncogene, *H19* can act as a tumor suppressor in select cancers such as retinoblastoma (Zhang et al., 2018). Due to its dual role, *H19* presents as a complex and compelling target for understanding cancer biology, determining prognosis, and developing effective treatment strategies for many types of cancers.

Disruptions to the imprinting balance between *H19* and *IGF2* due to LOI or aberrant DNA methylation have been observed in various cancers and are associated with biallelic expression of *IGF2* and suppression or reactivation of *H19*, leading to tumorigenesis (Ghafouri-Fard et al., 2020). The misregulation of these two genes can enhance proliferative signaling and lead to EMT, which highlights the role of *H19*/*IGF2* as a key modulator in cancer development.

Studies have shown that upregulation of *H19* is present in various cancer cell lines. Increased expression of lncRNA H19 was correlated with increased tumor size and advanced tumor node metastasis for non-small cell lung cancer (Liu et al., 2019). Silencing of *H19* in knock-down studies showed decreases in viability, invasion, and metastatic ability of cancer cells. Studies on thyroid cancer have shown varying roles of *H19*. While one study revealed the proliferative effects of *H19* by mediating the P13K/AKT pathway, another study showed that *H19* had a suppressive effect on cell migration and invasion in thyroid cancer cells (Ghafouri-Fard et al., 2020). The variable nature of *H19* in either suppressing or enhancing cell growth amongst and within various cancers makes it a unique target for oncologic therapies.

While *H19* functions through several target genes, one of the pathways impacted most by *H19* is the EMT mechanism in various cancers, including breast, bladder, and lung cancer (Liu et al., 2019). Mouse models of metastatic breast cancer have shown that lncRNA H19 acts as a sponge to sequester tumor suppressor *let-7b*, leading to the activation of ADP-ribosylation factor (ARF) proteins involved in cell migration linked to metastasis (Zhou et al., 2017). Sponging of *let-7b* by *H19* also showed a decrease in epithelial cadherin (E-cadherin), further contributing to the role of *H19* in inducing EMT (Zhou et al., 2017). Polymerase chain reaction (PCR) studies on *H19* in lung adenocarcinoma revealed that *H19* targets and downregulates the expression of miR-29b-3p, which regulates Signal Transducer and Activator of Transcription 3 (STAT3). Activation of STAT3 has been shown to play a role in cancer initiation and progression by increasing cell proliferation, decreasing apoptosis, and activating EMT-specific proteins, including lung and bladder cancer (Liu et al., 2019; Ghafouri-Fard et al., 2020). Additionally, metastatic tumors have an increased expression of *H19* compared to non-metastatic tumors (Zhou et al., 2017). Taken together, *H19*’s involvement in EMT and metastasis highlights its promising potential as a candidate for not only therapeutic targeting but also as a circulating biomarker with clinically actionable relevance for assessing EMT activity and metastatic risk.

While lncRNA H19 largely functions as an oncogene, leading to cell proliferation, it was shown to have the opposite effect in retinoblastoma. Gain-of-function assays revealed that *H19* inhibits cell proliferation and induces apoptosis in retinoblastoma by downregulating STAT3 (Zhang et al., 2018). The dual ability of *H19* to induce or inhibit cell proliferation based on the type of cancer makes it a unique target for novel therapies. Screening for lncRNA H19 may help determine cancer prognosis after initial diagnosis.

Although numerous studies have characterized the contrasting oncogenic and tumor-suppressive functions of H19, these context-specific outcomes likely reflect underlying differences in chromatin environment, enhancer accessibility, and competition among noncoding RNAs. In specific tissues, open enhancer landscapes and permissive chromatin states facilitate H19-driven expression of pro-growth targets, whereas in others, repressive chromatin or competing miRNA networks constrain its activity. This mechanistic variability underscores that H19’s role in tumorigenesis is not binary but instead shaped by developmental lineage, epigenetic context, and cellular signaling landscape. For instance, tissue-specific enhancer availability and epigenetic marks, including H3K27me3 and DNA methylation at the H19 differentially methylated region, can dictate whether H19 promotes or restricts EMT-related transcriptional programs (Kim et al., 2003; Zhao et al., 2016). Likewise, competitive interactions between H19-derived miR-675 and IGF1R signaling fine-tune growth responses, creating divergent phenotypes across cancers (Keniry et al., 2012; Liu et al., 2019; Zhou et al., 2017). Taken together, these findings suggest that the apparent contradictions in H19’s function stem less from inconsistency among studies than from its integration into dynamic chromatin and RNA regulatory networks that vary by developmental origin and tumor microenvironment.

### *IGF2* misregulation in cancer

Similarly to *H19*, LOI of *IGF2* has also been implicated in the development of cancer. IGF2 controls cell proliferation, apoptosis, and migration through regulation of the *Wnt3* gene (Boo et al., 2023). Biallelic expression of *IGF2* due to LOI leads to upregulation of *Wnt3* to stimulate cell proliferation. DNMT3A controls methylation of *IGF2*, and when overexpressed, DNMT3A leads to increased expression of *IGF2,* subsequently causing unrestrained proliferation through excess *Wnt3* signaling. While upregulation of *Wnt3* can lead to cancer, studies on lung tissue have shown that this upregulation of *Wnt3* can also be protective in scenarios of alveolar damage due to cigarette smoking (Boo et al., 2023). Chronic exposure to cigarette smoke damages alveoli and causes emphysema before tumorigenesis. So, overexpression of *IGF2* and *Wnt3* signaling can initially be beneficial for patients who chronically smoke, before becoming pathogenic for cancer. Mouse models showed that treatments that target DNMT or *IGF2* signaling can reduce cell proliferation and control tumor growth (Boo et al., 2023). However, *IGF2*’s protective role in repairing damaged lung tissue complicates the development of pharmacologic therapies that target *IGF2*. Further research that analyzes how to control the balance between *IGF2’*s protective and oncogenic roles in lung tissue should be conducted before *IGF2* can be seen as a promising target for cancer treatments.

### Wilms tumor and the 11p15 H19/IGF2 locus

Wilms tumor, nephroblastoma, is a prototypical embryonal cancer in which loss of imprinting (LOI) at 11p15, particularly biallelic IGF2 expression with concomitant dysregulation of H19, contributes directly to tumorigenesis (Xie et al., 2024; Sélénou et al., 2022). On a molecular level, focal hypomethylation within ICR1 unlocks access of shared enhancers to the IGF2 promoter on the maternal allele, amplifying IGF2 output and reactivating fetal growth programs in the metanephric blastema (Liao J et al., 2023). This mechanism sits squarely within the epigenetic framework we outline for imprint maintenance (DNMT1/UHRF1 propagation; DNMT3A/3B *de novo* establishment) (Liao W et al., 2023) and mirrors the developmental dosage logic we described previously (Table 3). Clinically, the tight association between Beckwith-Wiedemann syndrome (BWS) and Wilms tumor risk underscores the developmental-oncogenic spectrum at this locus: ICR1 hypermethylation on the maternal allele in BWS yields biallelic IGF2 and H19 silencing during development (overgrowth phenotype) and also predisposes to Wilms, illustrating how imprinting errors can manifest first as growth syndromes and later as embryonal tumors (Sélénou et al., 2022; Xie et al., 2024). Integrating Wilms tumor thus provides a concrete model in which imprinting failure at H19/IGF2 bridges embryogenesis and cancer, consistent with our comparative theme and the DNMT-linked mechanisms already cited (Liao J et al., 2023).

**TABLE 3:** Cancers with LOI at the IGF2/H19 locus and their developmental counterparts.

Cancer (embryonal/solid)	LOI/11p15 alteration at H19/IGF2	Developmentally linked tissue	Mechanistic connection
Wilms tumor (nephroblastoma)	ICR1 hypomethylation; biallelic IGF2; H19 dysregulation	Kidney (metanephric mesenchyme)	Reactivation of fetal growth programs; imprint failure aligns with embryonal histology
Hepatoblastoma	LOI with IGF2 overexpression	Fetal liver	IGF2-driven proliferation pathways parallel hepatic organogenesis
Adrenocortical carcinoma	Frequent IGF2 overexpression linked to 11p15 alterations	Adrenal cortex	Locus dysregulation mirrors steroidogenic fetal proliferation programs
Colorectal carcinoma	LOI of IGF2 documented in subsets	Intestinal epithelium	IGF axis re-engagement; developmental pathways re-purposed in tumorigenesis

This table consolidates malignancies with reported LOI/11p15 dysregulation, explicitly pairing each cancer to its developmental tissue of origin to match the review’s comparative frame. Mechanistic connections to imprint maintenance (DNMT/UHRF1) and to PI3K/AKT–MAPK, signaling are cross-referenced in the manuscript (Ren, 2022; Gicquel et al., 1997).

### Role switching of *H19* and *IGF2*: from embryogenesis to oncogenesis

The imprinted genes *H19* and *IGF2* are tightly coregulated during embryogenesis to control growth and differentiation. Under normal conditions, *H19* is expressed from the maternal allele, functioning primarily as a long-noncoding RNA that restricts growth. In contrast, *IGF2*, expressed from the paternal allele, acts as a potent growth-promoting factor (Xie et al., 2024). This reciprocal imprinting is maintained through epigenetic modifications at the ICR, which governs access to shared enhancers (Liao W et al., 2023). This tight regulation lays the groundwork for their opposing roles in development, where maintaining the right balance between *H19* and *IGF2* is essential for proper tissue growth.

During embryonic development, the opposing actions of *H19* and *IGF2* are essential for organogenesis. *IGF2* acts as a potent mitogen that drives cellular proliferation and tissue expansion, primarily through signaling pathways such as PI3K/AKT and MAPK, as demonstrated in both *in vitro* and *in vivo* models (Wang et al., 2018). At the same time, *H19* is implicated in growth restraint and developmental regulation, with overexpression leading to growth restriction and structural anomalies in multiple organ systems (Chang et al., 2022). The dynamic balance between these genes ensures the orderly progression of development. Although the precise mechanisms remain incompletely defined, *H19* has been proposed to exert its effects through microRNA regulation and chromatin-associated functions in other contexts. The dynamic interplay between *H19* and *IGF2* and the requirement for precise gene dosage underscores their critical role in coordinating developmental growth and differentiation (Chang et al., 2022; Wang et al., 2018).

**FIGURE 1:**
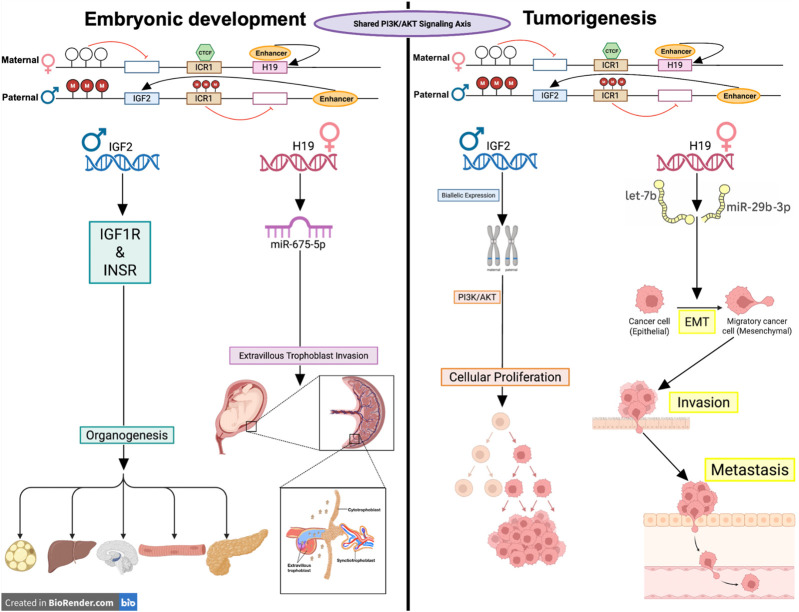
*H19/IGF2 locus as a shared molecular axis in embryogenesis and tumorigenesis.* This schematic illustrates how epigenetic regulation at the H19/IGF2 locus drives divergent outcomes in development and cancer via a shared PI3K/AKT signaling axis. Left panel: In embryogenesis, maternal ICR1 is unmethylated, allowing CTCF binding and promoting H19 expression while silencing IGF2. On the paternal allele, ICR1 is methylated, allowing IGF2 expression and silencing H19. IGF2 binds IGF1R and INSR receptors to drive PI3K/AKT signaling, facilitating organogenesis across multiple tissues, including adipose, liver, brain, muscle, and pancreas. Concurrently, H19, a long non-coding RNA, functions as a growth suppressor and contains an embedded microRNA, miR-675-5p, within its first exon. Post-transcriptionally, H19 produces miR-675-5p, which regulates extravillous trophoblast (EVT) invasion—an essential process for spiral artery remodeling and placental development. Right panel: In tumorigenesis, loss of imprinting (LOI) or methylation defects result in biallelic IGF2 expression and/or aberrant H19 reactivation. IGF2 overexpression amplifies PI3K/AKT activity, inducing unchecked cellular proliferation. H19 sponges tumor-suppressive miRNAs (let-7b and miR-29 b-3p), driving epithelial-to-mesenchymal transition (EMT), invasion, and metastasis.

However, this same growth-regulatory machinery becomes vulnerable when epigenetically dysregulated. Aberrant methylation at the *H19* DMR can disrupt the normal balance of *H19* and *IGF2* expression, leading to *IGF2* overexpression and *H19* repression, as observed following toxic metal exposures (Jo et al., 2024). In this oncogenic context, upregulation of *IGF2* enhances proliferative signaling pathways such as PI3K/AKT and MAPK, while dysregulation of *H19* can disrupt normal differentiation cues and promote metastasis (Jo et al., 2024; Wang et al., 2018). Thus, mechanisms initially designed to finely balance growth during embryogenesis can foster malignant transformation when disrupted. This dual role of the H19/IGF2 locus, promoting orderly growth during development and facilitating malignancy when dysregulated, is illustrated in Figure 1.

Mechanistically, overexpression of *IGF2* enhances survival signals and resistance to apoptosis, while dysregulated *H19* can function either as an oncogene or, paradoxically, as a tumor suppressor, depending on the context. This dual behavior of *H19* has sparked some controversy (Xie et al., 2024; Jo et al., 2024). Some studies report that elevated *H19* expression enhances tumor metastasis and stemness, particularly through modulation of EMT-related genes, while others suggest that loss of *H19* may also promote aggressive phenotypes. Based on current evidence, the prevailing view is that the role of *H19* is highly context-dependent, influenced by cancer type, microenvironment, and the relative balance of other regulatory RNAs and proteins. The variable nature of *H19* expression likely reflects its original developmental role in managing dynamic changes in gene expression during embryogenesis.

Moreover, the plasticity and rapid growth of embryonic tissues are typically the same features that tumor cells exploit during oncogenesis. Studies show that *H19* itself may act as a molecular sponge for tumor-suppressive miRNAs, thereby promoting stemness and invasiveness under pathological conditions (Xie et al., 2024). In this way, the developmental roles of *H19* and *IGF2* provide a blueprint for tumors to reactivate inappropriately for their expansion.

### Comparative insights: embryology versus cancer

Understanding the dual relationship between *H19* and *IGF2* and their roles in embryology compared to cancer offers significant insight into both developmental biology and oncology. From the embryological perspective, studies demonstrate that *H19* and *IGF2* are integral to controlling not just organ size but also the spatial-temporal patterning of tissues (Chang et al., 2022). Their precise temporal and spatial expression is required to form complex structures such as the heart and placenta properly. The strict parent-of-origin-specific expression of these genes ensures that maternal and paternal genomes contribute asymmetrically to growth regulation, protecting against aberrant development.

Even during fetal development, aberrant expression of *H19* and *IGF2* can lead to severe developmental syndromes. As discussed earlier, syndromes such as BWS and SRS highlight how disruptions in the ICR of *H19*/*IGF2* during fetal development can lead to placental overgrowth or growth restriction, respectively (Liao J et al., 2023; Chang et al., 2022). These clinical observations demonstrate the importance of imprinting balance for normal human development (Chang et al., 2022). Insights into these regulatory mechanisms not only enhance our understanding of normal development but also provide crucial context for understanding how LOI or dysregulation of these genes can predispose to malignancy later in life.

Conversely, in oncology, misregulating these same imprinted domains illustrates how cancers can co-opt developmental programs. Overexpression of *IGF2* drives unchecked proliferation and survival, while altered *H19* expression can promote EMT and metastasis (Jo et al., 2024). By examining how tumors reactivate embryonic growth mechanisms, deeper insights into cancer progression can be achieved, revealing novel therapeutic targets, particularly within epigenetic regulatory pathways.

Recent work has revealed additional layers of regulation within the IGF signaling pathway mediated by microRNAs which could be a potential therapeutic target. Notably, *miR-675*, derived from the imprinted *H19* transcript, directly targets *IGF1R* (Keniry et al., 2012), attenuating IGF signaling. This connection links the *H19/IGF2* imprinted locus to modulation of IGF receptor activity, reinforcing its central role in balancing growth and proliferation during development and tumorigenesis. *H19* and its derivative miRNA are part of a regulatory network that modulates cellular responses to growth signals. During embryogenesis, *H19* expression typically restrains excessive proliferation by limiting IGF1R-mediated signaling, whereas in cancer, reactivation of the *H19/miR-675* axis can either suppress or promote tumor growth depending on cellular context and receptor balance. This dual behavior highlights the intricate control exerted by imprinted loci over growth-promoting pathways.

Similarly, *miR-483*, which is encoded within the *IGF2* gene itself, represents another example of how imprinted genes regulate their own signaling environment. *miR-483* has been shown to target both *IGF1* (Ni et al., 2013; Sandovici et al., 2024) and *IGF2* (Liu et al., 2013). This creates a feedback loop in which *IGF2* expression drives IGF signaling and produces a microRNA capable of modulating IGF ligand abundance. These interactions highlight how microRNA-mediated regulation adds an additional layer of control to the IGF network, with potential implications for both normal developmental processes and the dysregulated growth characteristic of cancer. Disruption of these finely tuned mechanisms through altered imprinting, aberrant miRNA expression, or receptor overactivation may represent a common route toward uncontrolled proliferation and tumor development.

Interestingly, recent studies suggest that targeting epigenetic regulators of the *H19*/*IGF2* locus could restore normal imprinting and suppress tumorigenesis (Jo et al., 2024). This offers exciting therapeutic potential, as epigenetic drugs may reverse pathological gene expression without permanently altering the genome. The overall comparative analysis of these genes across development and disease highlights the fragility of epigenetic regulation. It emphasizes that proper growth depends not solely on gene presence but also on the precise context, timing, and regulation of gene expression.

### Clinical implications of *H19* and *IGF2* LOI

As continued research on the genetic aspects of cancer is conducted, the roles of epigenetic modifications in tumorigenesis are becoming more apparent (Oyon et al., 2025). As a result, there has been a surge in the development of epigenetic therapies, a treatment type that involves using pharmaceutical interventions to epigenetically regain control of aberrant gene expression in cancerous cells, often targeting enzymes such as histone deacetylases (HDACs) (Wang et al., 2024). *H19* and *IGF2* are both believed to play a significant role in EMT and tumor metastasis, making them prime targets for potential cancer treatments (Jo et al., 2024). Due to the sharp decline in patient prognosis associated with tumor metastasis, epigenetically modifying the expression of *H19* and *IGF2* in tumors and preventing further tumor invasion could drastically improve patient outcomes (Xu et al., 2021).

While clinical trials for epigenetic therapies are still in early phases, promising findings have already emerged from *in vitro* and animal model studies. In a 2024 study testing combination therapies, histone deacetylase inhibitors (HDACi), when used as a complementary therapy, showed therapeutic effects in colorectal cancer patients. The results of this study suggest that HDACis could be beneficial in cancers with LOI of *H19* and *IGF2,* given to the role of HDACs in downregulating gene expression (Wang et al., 2024). In an *in vitro* study, CM272, a drug that dually inhibits DNMT1 and the euchromatic histone-lysine methyltransferase G9a, improved the response of pancreatic ductal adenocarcinoma (PDAC) cells to chemotherapy. Additionally, it reduced the tumorigenic effects of both human and mouse PDAC cell lines (Oyon et al., 2025). Given the preliminary success of epigenetic therapies in other cancers, similar mechanisms could be employed in the epigenetic regulation of tumors with LOI of *H19* and *IGF2* specifically. DNMT3A and DNMT3B, as discussed earlier, are both involved in the developmental epigenetic regulation of *H19*, and drugs affecting their activity could be used for targeted cancer treatments (Han et al., 2023). It is believed that decreased activity of these DNA methyltransferases is associated with reduced *H19* expression, suggesting that drugs inhibiting DNMT3A and DNMT3B could epigenetically drive a decrease in *H19* expression (Han et al., 2023).

For these epigenetic treatments to become a standard of care, these epigenetic regulatory mechanisms need to be specified to the *H19/IGF2* loci. Luckily, recent studies have begun targeting and epigenetically modifying the *H19* and *IGF2* genes specifically. A 2024 study using HEK293 cell lines demonstrated stable demethylation of the *H19/IGF2* ICR1 (Albrecht et al., 2024). Using the dCas9-SunTag system and the catalytic domain of TET1, the epigenetic modifications remained stable throughout all 27 days of observation*. H19* expression levels remained increased throughout the entire observation period, though the *IGF2* expression levels were less stable, returning to control levels by the end of the period. In another study conducted in 2022, researchers used a similar system, the dCas9-SunTag and *piggyBac* transposons, and induced *H19* demethylation patterns in transgenic mice (Horii et al., 2022). The transgenic mice showed changes in both *H19* and *IGF2* expression levels, along with phenotypic changes consistent with Silver-Russell syndrome, a genetic disease characterized by *H19* LOI and demethylation. While these studies take promising steps toward developing epigenetic therapies specific to the *H19/IGF2* loci, they still lack the selectivity for cancer cells required to make this drug more clinically beneficial.

The burgeoning field of immunotherapy and antibody-drug conjugates (ADCs) could help bridge this gap in therapeutic utility. ADCs are a novel pharmaceutical technology that uses monoclonal antibodies conjugated with drugs to deliver targeted cytotoxic treatments (Wang et al., 2025). Harnessing the specificity of ADCs could help prevent the potentially injurious off-target effects of epigenetically altering non-cancerous cells in the body, though the method is not without faults (Zhang et al., 2024). One major issue facing both epigenetic therapies and combined epigenetic ADCs is tumor heterogeneity. Epigenetic therapy, in particular, requires extensive knowledge of the genetic and epigenetic landscape of each tumor to determine a patient’s eligibility for epigenetic therapy. Current genetic testing relies primarily on PCR technology, which is not always entirely accurate and can cost patients thousands of dollars (Vanderpoel et al., 2022; Xie et al., 2024). Additionally, ADCs conjugated with epigenetic therapies would take a significant amount of time to develop and pose serious adverse effects, including thrombocytopenia, anemia, and peripheral neuropathy (Jia et al., 2024).

Despite needing further development before becoming standard care, epigenetic therapies targeting the LOI in *H19* and *IGF2* can potentially revolutionize cancer therapies. Most importantly, there needs to be improvements in the speed and reliability of genetic testing, along with mechanistic changes in medications that preferentially target cancerous cells. With the role that *H19* and *IGF2* play in cancer EMT, it is plausible that inhibiting their imprinting misregulation could help prevent further invasion and metastasis of tumor cells. Additionally, the complementary use of *H19* epigenetic therapies could help improve the effectiveness of other treatments. *H19* overexpression has been linked to resistance against the chemotherapy drug cisplatin (Xu et al., 2021). It is believed that LOI in certain cancer cells could be partially behind the mechanisms of drug resistance, which is why reversal of the LOI could consequently help improve responsiveness to anti-cancer drugs (Oyon et al., 2025). Metastasis is one of the greatest indicators of a poor prognosis in multiple cancers, making it imperative that further research is conducted into treatment options such as epigenetic therapies that have the potential to slow, if not completely prevent, metastasis.

## Conclusion

The literature reveals that the *H19*/*IGF2* locus is not only a tightly regulated epigenetic unit essential for balancing fetal growth but also plays a key developmental role in integrating trophoblast function, organogenesis, and morphogenic transitions. Through parent-of-origin methylation patterns and coordinated PI3K/AKT signaling, *H19* and *IGF2* mediate proliferative and invasive cellular behaviors critical for early embryonic and placental development. Understanding the regulatory complexity of this locus reveals its broader function in ensuring developmental success.

The comparative analysis of *H19* and *IGF2* across embryogenesis and tumorigenesis highlights the remarkable duality of these imprinted genes. Mechanisms that ensure balanced growth and differentiation during development become oncogenic when dysregulated, with *IGF2* driving proliferation and *H19* variably promoting or suppressing tumor progression, depending on the context. This shift in functional roles emphasizes the intrinsic variability of epigenetic regulation at the *H19*/*IGF2* locus. Tumors exploit the same dynamic dosage sensitivity that regulates organ development to drive uncontrolled proliferation, metastasis, and cellular survival. Recognizing this overlap reveals not only the vulnerabilities of epigenetic control systems but also their therapeutic promise. As our understanding of imprinting mechanisms advances, the *H19*/*IGF2* relationship emerges as a compelling model for investigating how developmental pathways are reactivated in cancer and as a favorable avenue for epigenetic-based therapies.

Epigenetic therapies have the potential to change how cancers are treated by using pharmaceutical interventions to change the epigenome of dysregulated genes. Current research has shown promising results with epigenetic therapies, such as CM272, when combined with other cancer treatments, though there are still issues with a lack of cell-type and locus specificity. *H19* and *IGF2* are two genes where LOI in tumor cells is linked with poor prognoses and chemotherapy resistance, and being able to regain control of their expression could help improve patient outcomes and increase the effectiveness of additional treatments. Understanding the roles of *H19* and *IGF2* in embryology has helped researchers understand what mechanisms can be targeted for epigenetic therapies, highlighting the importance of embryology research to the development of cancer treatments.
